# A Machine Learning Framework for Preeclampsia Prediction at Isidro Ayora Hospital, Ecuador

**DOI:** 10.3390/diagnostics16142147

**Published:** 2026-07-08

**Authors:** Maria Perez, Lenin G. Falconi, Monserrate Intriago-Pazmiño, Andrés Bastidas-Fuertes, Juan Benavides, Raysa A. Fuertes-Arévalo

**Affiliations:** 1 Departamento de Informática y Ciencias de la Computación, Escuela Politécnica Nacional, Quito 170525, Ecuador; maria.perez@epn.edu.ec (M.P.); lenin.falconi@epn.edu.ec (L.G.F.); monserrate.intriago@epn.edu.ec (M.I.-P.); juan.benavides@epn.edu.ec (J.B.); 2 Smartwork S.A., Quito 170503, Ecuador; andres.bastidas@smartwork.com.ec; 3 Ministerio de Salud Pública, Quito 170702, Ecuador; raysafuertesa@gmail.com

**Keywords:** machine learning, explainable artificial intelligence, preeclampsia, prediction, LightGBM, ensemble models

## Abstract

**Background:** Preeclampsia (PE) is a leading cause of maternal and perinatal morbidity, affecting 2–8% of pregnancies worldwide. In Ecuadorian public hospitals, clinical data are often fragmented, limiting the construction of complete datasets for prediction modeling. This study aimed to develop a Machine Learning (ML) framework to predict PE using demographic data from the Isidro Ayora Gynecology and Obstetrics Hospital (HGOIA), Quito, Ecuador. **Methods:** We proposed a custom automated Machine Learning (C-AutoML) workflow to evaluate six models (Logistic Regression, LightGBM, XGBoost, CatBoost, Multi-Layer Perceptron, and Random Forest) and compared its performance against a weighted ensemble built with Amazon SageMaker Canvas, optimizing for the F1-score. Model interpretability was analyzed using Shapley Additive Explanations (SHAP). Due to the scarcity of clinical variables at HGOIA, a synthetic dataset based on FullPIERS predictors was also generated and modeled. **Results:** On the hospital dataset, the SageMaker ensemble achieved an F1-score of 0.805, accuracy of 0.934, and AUC of 0.956, while the C-AutoML best single model (LightGBM) yielded comparable results (F1-score: 0.802, accuracy: 0.938, AUC: 0.944). SHAP analysis identified patient age as the most influential feature. On the synthetic dataset, Logistic Regression achieved perfect classification (F1-score: 1.0). **Conclusions:** The proposed C-AutoML framework matched the performance of the SageMaker weighted ensemble while relying on a single, interpretable model. Despite the current dependence on demographic data, the methodology shows promise for supporting PE diagnosis in resource-limited settings; future integration of clinical variables is expected to further enhance predictive power for risk stratification.

## 1. Introduction

Preeclampsia (PE), a leading hypertensive disorder of pregnancy, is a major cause of maternal and perinatal morbidity and mortality worldwide, being associated with more than 70,000 maternal deaths and over 500,000 fetal and neonatal deaths annually [1]. The World Health Organization (WHO) reports that PE affects approximately 2–8% of pregnancies globally, with a pooled global prevalence estimated at 4.43% [1], highlighting the relevance of early detection and risk stratification strategies [1,2].

Clinically, PE is a complex multisystem disorder defined as new-onset hypertension (Systolic Blood Pressure (SBP) ≥ 140 mmHg or Diastolic Blood Pressure (DBP) ≥ 90 mmHg) after 20 weeks of gestation in previously normotensive women, accompanied by at least one of: proteinuria, evidence of maternal end-organ dysfunction, or signs of uteroplacental insufficiency such as fetal growth restriction. This departs from earlier criteria that required proteinuria in all cases [1,3]. The condition is multifactorial in origin, with key risk factors including advanced maternal age, obesity, chronic hypertension, diabetes, and limited access to prenatal care [1]. Although PE typically develops in the second half of pregnancy, clinical onset can occur for the first time during labor or within the early postpartum period, as the underlying vascular dysregulation does not resolve immediately at delivery [3]. International guidelines further distinguish early-onset PE (onset before 34 weeks of gestation) from late-onset PE (at or after 34 weeks), which differ in placental pathophysiology, severity profile, and associated neonatal morbidity [3]. Contemporary practice discourages classifying cases into separate mild and severe forms, as even apparently moderate presentations may progress abruptly to life-threatening complications; presentations accompanied by markedly elevated blood pressures, thrombocytopenia, renal insufficiency, hepatic dysfunction, pulmonary edema, or new-onset headache are instead characterized as having severe features, requiring urgent management [3]. Collectively, these features underscore the clinical complexity of PE and the need for robust, population-adapted analytic strategies to support timely identification of at-risk pregnancies [1].

In Ecuador, the Clinical Practice Guideline from the Ministerio de Salud Pública (MSP) (Ministry of Public Health) identifies PE and eclampsia among the principal causes of maternal death. It reports that these conditions were the leading cause of maternal mortality, accounting for 27.53% of maternal deaths from 2006 to 2014 [4]. This epidemiological burden supports the need for local analytic approaches that characterize risk patterns in hospital populations and can inform timely referral and surveillance.

In recent years, Machine Learning (ML) has been increasingly applied to PE prediction, with studies showing promising discriminatory performance using routinely collected variables [5,6,7,8]. These approaches identify non-linear risk patterns from maternal characteristics, vital signs, laboratory measurements, and obstetric history, enabling earlier and more individualized risk estimation than rule-based screening alone [5,6]. Clinically, this is important for proactive surveillance, prioritization of high-risk pregnancies, and more efficient allocation of limited healthcare resources, particularly in high-volume settings with heterogeneous data quality.

The full Preeclampsia Integrated Estimate of RiSk (PIERS) model estimates the probabilistic risk of adverse maternal outcomes in women with PE using Gestational Age (GA), chest pain or dyspnoea, oxygen saturation (${\mathrm{SpO}}_{2}$), creatinine, platelet count, and aspartate transaminase (Aspartate Aminotransferase (AST)), including quadratic and interaction terms, as predictors [9]. It uses the Logistic Regression equation in (1), where the logit is calculated as a linear combination of these variables as depicted in (2) and the coefficient values are those proposed in [9].

(1) $$\mathrm{Probability}\;\mathrm{Risk}\left(\mathrm{logit}\right)=1/\left(1+\exp^{-\mathrm{logit}}\right)$$

(2) $$\begin{array}{cc} \mathrm{logit}(\pi ) & =2.68\\ \; & \;+(-5.41\times 10^{-2}\times \mathrm{gestational}\;\mathrm{age}\;\mathrm{at}\;\mathrm{eligibility})\\ \; & \;+(1.23\times \mathrm{chest}\;\mathrm{pain}\;\mathrm{or}\;\mathrm{dyspnoea})\\ \; & \;+(-2.71\times 10^{-2}\times \mathrm{creatinine})\\ \; & \;+(2.07\times 10^{-1}\times \mathrm{platelets})\\ \; & \;+(4.00\times 10^{-5}\times {\mathrm{platelets}}^{2})\\ \; & \;+(1.01\times 10^{-2}\times \mathrm{aspartate}\;\mathrm{transaminase})\\ \; & \;+(-3.05\times 10^{-6}\times {\mathrm{AST}}^{2})\\ \; & \;+(2.5\times 10^{-4}\times \mathrm{creatinine}\times \mathrm{platelet})\\ \; & \;+(-6.99\times 10^{-5}\times \mathrm{platelet}\times \mathrm{aspartate}\;\mathrm{transaminase})\\ \; & \;+(-2.56\times 10^{-3}\times \mathrm{platelet}\times {\mathrm{SpO}}_{2}) \end{array}$$

However, a major operational challenge for Electronic Health Records (EHR) in Ecuador is the fragmentation of clinical information systems. In many hospitals linked to the MSP, complementary examinations are recorded in separate databases or remain only in paper-based formats, which limits immediate construction of complete and standardized electronic clinical datasets for modeling. As a consequence, in this research, we used the provided records from the Isidro Ayora Gynecology and Obstetrics Hospital (HGOIA) (Hospital Gineco Obstétrico Isidro Ayora (HGOIA), a third-level public specialty hospital in Zone 9 of the MSP, Quito, Ecuador), which contains demographic variables and lacks clinical features. For this reason, we explored our sets of models both on the HGOIA and synthetic datasets to diagnose PE. The synthetic dataset was constructed using the fullPIERS Equation (2) as the reference framework for feature distributions and the MSP clinical guideline [4] for the diagnostic label (Section 3.5). Unlike the fullPIERS standard, which is a prognostic model, our goal is to determine the most effective set of models to diagnose PE according to the Ecuadorian realm.

In this work, we propose a custom automated Machine Learning workflow that explores up to six classification algorithms (Logistic Regression (LR) [10], LightGBM [11], XGBoost [12], CatBoost [13], Multi-Layer Perceptron (MLP) [14], and Random Forest (RF) [15]) as candidate models for PE diagnostic prediction. Amazon SageMaker was also used to evaluate the predictive power of the demographic variables of the dataset of patients at HGOIA and to train a reference classifier. To improve classification, given the imbalanced data, we target the F1-score (F1) metric to balance sensitivity and precision. Our results show that an ensemble model formed by LightGBM, RF and MLP achieves F1 of 0.805, outperforming single models. To provide interpretability for our model and avoid its use as a black-box, we employed SHapley Additive exPlanations (SHAP) to quantify the contribution of each feature to the model’s predictions. Patient age is the most influential feature followed by the GA in weeks. Finally, to account for the lack of clinical data records, we explored the same models with a synthetic dataset of our own.

The contributions of our work are as follows: (i) to the best of our knowledge, this is the first study to address the use of machine learning algorithms in PE in Ecuador, (ii) we systematically explored different Machine Learning models aiming to improve classification performance as well as model’s explainability, (iii) we used data provided from HGOIA, a specialized public health facility in gynecology and obstetrics, (iv) we explored additional models on synthetic data to account for lack of clinical variables, (v) we deployed our model using a Machine Learning Operation (MLOP) approach in a server at Escuela Politécnica Nacional (EPN), including a web-based inference interface (see Appendix B).

## 2. Related Works

Recent literature shows sustained interest in using ML for PE prediction, but also highlights important differences in study design, data sources, and intended clinical use. Two recent systematic reviews synthesize this landscape. Darsareh et al. [7] reviewed ML-based prediction studies and reported that the best-performing approaches commonly included elastic net, stochastic gradient boosting, extreme gradient boosting, and Random Forest, with Area Under the Receiver Operating Characteristic Curve (AUC) values ranging approximately from 0.860 to 0.973. Similarly, Rahman et al. [8] surveyed studies published between 2018 and 2022 and concluded that ML was the dominant analytical paradigm in this domain, with ensemble and boosting methods frequently achieving the highest predictive performance. Their review also shows that demographic variables were used much more frequently than laboratory variables among the sampled papers, with demographic features representing 53% of the reviewed inputs versus only 15% for laboratory data [8]. This observation is relevant in our context, given the type of variables gathered from HGOIA for our study. However, we disagree with their model ranking, which primarily emphasizes accuracy, because from a clinical point of view, overlooking high-risk pregnancies is more consequential than accepting a moderate increase in false positives. Hence, we consider the ranking should be on the F1 or recall metrics, since a higher value in recall, reduces the false negatives.

Beyond systematic reviews, several primary studies illustrate the diversity of PE prediction strategies. Marin et al. [16] used the Viterbi algorithm to determine the presence of PE based on features such as the weight, age, and blood-pressure measurements. Their approach was integrated with a smart bracelet and a mobile application to monitor the evolution of the physiological parameters. This showed potential to be used in hospitals as well as in remote monitoring of patients for early detection of the condition. Li et al. [5] explored LR, RF, Support Vector Machines (SVMs), and XGBoost models to predict women at risk of PE from Electronic Health Records collected in early pregnancy. The XGBoost model achieved the best performance (ACC=0.920, Precision=0.447, Recall=0.789, F1=0.571, AUC=0.955) and showed that routinely available structured clinical variables combining maternal factors and laboratory data, such as fasting plasma glucose, mean blood pressure, and Body Mass Index (BMI), can provide useful discriminative power for PE risk prediction. Marić et al. [17] used Elastic Net Logistic Regression (EN) with stacked multiomics integration on longitudinal data from a small prospective pregnancy cohort and achieved early prediction of PE (AUC=0.83) using a 9-metabolite urine model, with performance improving to AUC=0.94 when integrating multiple omics modalities, demonstrating that molecular features are strong contributors to predictive performance because they capture pathophysiological processes before symptom onset and change earlier than traditional clinical signs; clinical variables were predictive but secondary and complementary, providing less information than omics-derived biomarkers.

Taken together, prior work suggests three major trends. First, PE prediction models tend to perform best when they combine multiple domains of information, including maternal history, hemodynamic measurements, laboratory values, and sometimes ultrasound or omics data [6,7,8]. Second, tree-based ensembles and boosting algorithms are frequently reported as top performers, although simpler models such as logistic regression or elastic net can remain competitive when predictors are well structured and clinically meaningful [5,7,18]. Third, despite strong reported predictive metrics, many studies were developed in data-rich environments and depend on comprehensive electronic records that may not be available in middle-income public hospital settings.

Our study is positioned within this literature but addresses a different operational context. Instead of assuming a mature, fully integrated electronic clinical infrastructure, we combine: (i) a descriptive analysis of retrospective patient records from HGOIA, (ii) a cloud-managed benchmarking workflow, and (iii) a custom automated ML pipeline with explainability analysis. In addition, because fragmented records currently limit access to a fully standardized clinical prediction dataset, we include a synthetic-data track for controlled methodological exploration. This makes our work complementary to prior studies by focusing not only on predictive performance, but also on the practical transition path toward local, interpretable PE modeling in an operational public-hospital environment.

## 3. Materials and Methods

In this section, we describe our methodological approach to diagnose PE in women at the Isidro Ayora Gynecology and Obstetrics Hospital (HGOIA). We restricted our prediction to diagnosis due to the limitations of the provided data. Our research problem is stated in Equation (3), where given a feature vector $x\in R^{d}$ representing attributes of a pregnant woman, the probability that a patient has PE is modeled as

(3) P(PE=1∣x)=f(x;θ)

where $f:R^{d}\to \left[0,1\right]$ is a prediction function parameterized by θ. For a binary classifier, the final prediction $\hat{y}$ is

(4) $$\hat{y}=\left\{\begin{array}{cc} 1 & \mathrm{if}\;P(\mathrm{PE}=1|x)\geq \tau \\ 0 & \mathrm{otherwise} \end{array}\right.$$

with τ∈[0,1] being a decision threshold, typically τ=0.5.

### 3.1. Study Design

This was a descriptive, cross-sectional observational study that used an anonymized database of medical records from patients at the HGOIA in Quito, Ecuador. Our research goal is to train a Machine Learning classification model to predict PE. We explore 6 different classification models to improve performance. Explainability is addressed for each model to show the influence of a given feature $x_{i}$ in the diagnostic prediction. The study was approved by the Research Ethics Committee for Human Subjects of Pontifical Catholic University of Ecuador (PUCE) (CEISH-PUCE), under protocol code EO-086-2024 (version 4), approval number CEISH-281-2025, on 23 April 2025.

### 3.2. Data Collection

The records provided by HGOIA contain a total of 174,753 records and 49 descriptive variables (features) from year 2021 to 2025, accessed under the ethical approval granted by CEISH-PUCE (code CEISH-281-2025). However, the dataset contains information about all the different diseases treated at the hospital according to International Classification of Diseases (ICD-10).

### 3.3. Data Preprocessing

**Figure 1:**
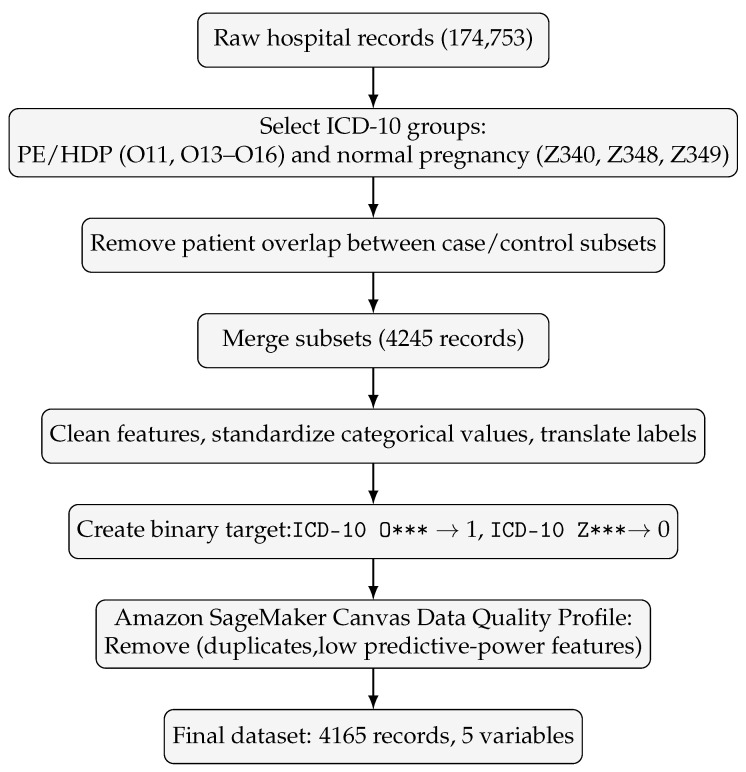
Preprocessing workflow used to construct the HGOIA preeclampsia classification dataset. Z*** and O*** denote ICD-10 codes selected for this research where each * denotes a digit following the category letter.

Data preprocessing was designed to construct a reliable binary classification dataset for PE prediction from routine hospital records. Figure 1 summarizes the complete workflow.

**Figure 2:**
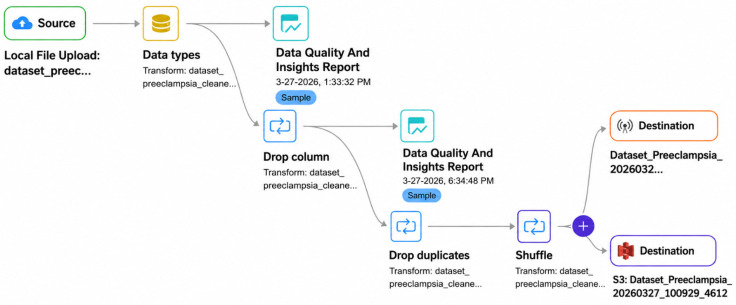
Data Wrangler is used to assess the quality of data before training the Machine Learning model. Low predictive power columns and duplicated records were removed. Resulting data is shuffled to prevent data leakage.

- Case identification: We selected records associated with hypertensive disorders in pregnancy using MSP guidance and ICD-10 codes O11, O13, O14, O15, and O16. From 174,753 records, 674 were identified as PE-related cases. Table 1 describes the codes selected.
- Control identification: We selected normal pregnancy records using ICD-10 codes Z340, Z348, and Z349, obtaining 3667 records. Table 1 describes the codes selected.
- Overlap removal: Patients appearing in both groups were removed from the normal pregnancy subset to avoid label leakage, reducing the control group to 3571 records.
- Dataset assembly: The PE and normal pregnancy subsets were merged into a single dataset with 4245 records.
- Feature cleaning and harmonization: Variables with excessive missingness were removed (49 to 22 original predictors), and categorical values were standardized and translated to English.
- Target encoding: A binary outcome variable was created by assigning a value of 1 to records with ICD-10 codes beginning with O and 0 to records with codes beginning with Z. This resulted in a total of 3571 normal pregnancies and 674 PE cases, as summarized in Table 1.
- Data quality profiling in Amazon SageMaker Canvas: We used Data Wrangler in Amazon SageMaker AI to assess data quality. The profiling report identified duplicated records and performed a univariate predictive evaluation of each feature. A duplicate rate of 1.84% was detected and manually confirmed. We then removed predictors with zero predictive power, according to the profiling report. Figure 2 shows the quality operations performed in Data Wrangler. Table 2 summarizes the main dataset-quality outputs reported by Amazon SageMaker Canvas.
- Final dataset: After duplicate removal, feature filtering, and shuffling, the final dataset contained 4165 records and 5 variables (including the target, PREECLAMPSIA). These variables are: maternal age (years), GA (weeks), BMI ($\mathrm{kg}/m^{2}$), and weight (kg). Table 3 presents a summary of the HGOIA dataset characteristics at each stage of the preprocessing pipeline.

### 3.4. Statistical Characteristics of Collected Data

**Figure 3:**
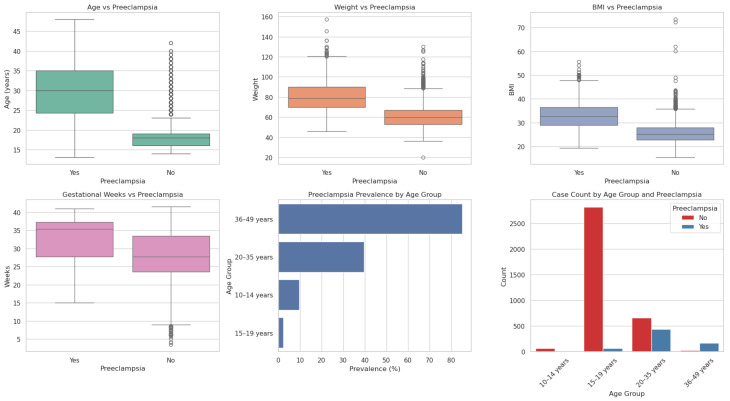
Statistical analysis of prevalence of preeclampsia in the population of pregnant women at HGOIA. Preeclampsia is more frequent in women of 36 to 49 years old and with a mean BMI of 33. Analysis based on the pre-deduplication cohort (4245 records: 3571 normal, 674 preeclampsia; see also Table 3).

Table 4 presents statistical characteristics of demographic variables in the dataset, whereas Table 5 shows the distribution of those variables organized in quartiles with respect to (w.r.t.) the prevalence of PE in pregnancies at HGOIA. We analyzed the prevalence of PE among the population of pregnancies at the dataset provided by HGOIA. Some of the important insights are that PE prevalence rises strongly with age, reaching 85.13% in the 36–49 year group (Table 5). Women diagnosed with PE have a mean BMI of 33. Normal pregnancies have a mean BMI of 25.71. These results are summarized in Figure 3.

The record-level prevalence of PE in the final dataset is 16.11% (671 of 4165 records), exceeding the global pooled estimate of 4.43% [1]. This reflects two factors: the dataset was constructed from a case-enriched selection of ICD-10 codes (Section 3.3) rather than the full obstetric population, and HGOIA is a specialized referral centre that receives high-risk pregnancies directed by the MSP. As a result, the reported precision and F1 are calibrated to this cohort prevalence and would decrease in lower-prevalence settings. Table 4 and Table 5 provide inter-group comparisons of all model input variables between PE-positive and PE-negative groups; all four predictors show highly significant differences (Mann–Whitney *U* and Pearson $\chi^{2}$, p<0.001 for all variables), confirming their discriminative value within this cohort.

### 3.5. Synthetic Data Generation

To complement the retrospective demographic dataset from HGOIA and compensate for limited availability of structured clinical variables, we generated a synthetic dataset designed to simulate clinical data profiles for pregnant patients with and without PE. The generation process was implemented in Python (3.9.23) using NumPy (2.0.2) and pandas (2.3.1), with a fixed random seed for reproducibility.

The algorithm generates n=2000 samples (default: n=1000) and first assigns each sample to one of two latent groups: Control (80%) or Risk (20%). sampled from predefined distributions chosen to reflect expected clinical behavior. The generated variables are maternal age (Age), GA in weeks (${\mathrm{GA}}_{\mathrm{wk}}$), systolic and diastolic blood pressure (SBP, DBP), 24 h proteinuria, oxygen saturation (${\mathrm{SpO}}_{2}$), platelet count (Plt), creatinine (μmol/L), AST, and a chest pain indicator. Table 6 presents the statistical distributions (N,Gamma,Bernoulli) used for the generation of each variable. Negative values were avoided by clipping.

After feature generation, a binary diagnostic label (PE) is assigned using a simplified rule based on the Ecuadorian MSP clinical guideline for Hypertensive Disorders of Pregnancy (HDP) [4], where Hypertension (HTN) denotes hypertension and Upper Limit of Normal (ULN) denotes the upper limit of normal for laboratory values. Let HTN=(SBP≥140)∨(DBP≥90), $\mathrm{Prot}=({\mathrm{Prot}}_{24h}\geq 300\;\mathrm{mg})$, $\mathrm{TCP}=(\mathrm{Plt}<100\times 10^{9}\;L^{-1})$, $\mathrm{Renal}=({\mathrm{Cr}}_{\mathrm{mg}/\mathrm{dL}}>1.1)$, and Hep for hepatic involvement defined as AST≥2×ULN with ULN=40U/L. Then,

(5) $${\mathrm{PE}}_{\mathrm{MSP}}=1\Leftrightarrow \left({\mathrm{GA}}_{\mathrm{wk}}\geq 20\right)\wedge \mathrm{HTN}\wedge \left(\mathrm{Prot}\vee \mathrm{TCP}\vee \mathrm{Renal}\vee \mathrm{Hep}\right).$$

In addition to the binary diagnostic label, a continuous prognostic risk score was computed using the published fullPIERS model [9]. For each sample, the algorithm computes a logit function of GA, chest pain, ${\mathrm{SpO}}_{2}$, Plt, AST, Cr, and their interaction terms, and then transforms it into a probability according to (1) and (2). The final synthetic dataset therefore contains both a rule-based diagnostic outcome (${\mathrm{PE}}_{\mathrm{MSP}}$, defined in Equation (5)) and a continuous prognostic risk estimate (${\mathrm{PE}}_{\mathrm{FullPIERS}}$ [%]), enabling exploratory modeling and explainability analyses under clinically informed constraints.

Although the ${\mathrm{PE}}_{\mathrm{FullPIERS}}$ score was used to obtain an exploratory binary label with a threshold of 10%, empirical validation studies have reported optimal cut-offs between 2 and 6% in different populations [19,20,21]. For the diagnostic task on the synthetic dataset, however, the primary classification target for model training and evaluation is the rule-based label ${\mathrm{PE}}_{\mathrm{MSP}}$ defined in (5), which does not depend on ${\mathrm{SpO}}_{2}$ nonetheless retained in the synthetic dataset because they are validated independent predictors of adverse maternal outcomes in women with established preeclampsia [21,22].

### 3.6. Machine Learning Models and Training

This research aims to diagnose PE with the provided data from HGOIA. In Section 3, we defined the PE diagnostic problem as a binary classification. Consequently, this study used two complementary training tracks: (i) a cloud-managed workflow in Amazon SageMaker Canvas for rapid no-code benchmarking, and (ii) C-AutoML for controlled experimentation and methodological traceability (see Appendix A).

#### 3.6.1. Model Families

We evaluated six supervised model families commonly used in binary classification, including gradient-boosted decision trees (XGBoost, LightGBM, and CatBoost), RF, MLP, and regularized LR.

- Logistic Regression (LR): A linear probabilistic baseline with high interpretability and robust behavior in tabular clinical settings [10].
- Random Forest (RF): A bagging-based tree ensemble that captures non-linear interactions and is robust to noisy predictors [15].
- Multi-Layer Perceptron (MLP): A feed-forward model able to represent higher-order non-linear relationships [14].
- XGBoost: Gradient-boosted decision trees with regularization and optimized implementation for strong predictive performance on structured data [12].
- LightGBM: A histogram-based gradient boosting framework designed for computational efficiency and scalability on large tabular datasets [11].
- CatBoost: An ordered boosting method with native support for categorical variables and reduced prediction shift [13].

#### 3.6.2. Training Process

**Figure 4:**
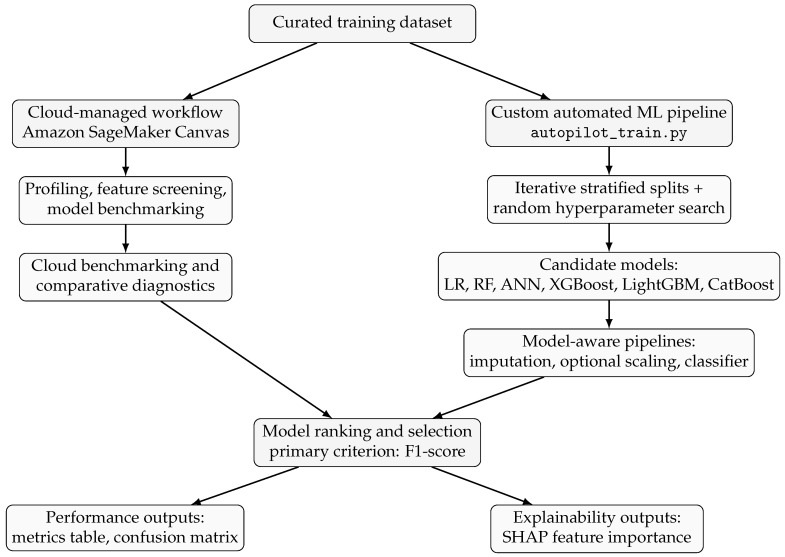
Proposed Machine Learning training framework combining cloud-managed (SageMaker Canvas) and a custom automated ML pipeline for model development, selection, performance evaluation, and SHAP-based explainability.

Figure 4 illustrates the proposed Machine Learning training framework used in this study, including the parallel cloud-managed and script-based workflows.

##### Cloud-Managed Training (Amazon SageMaker Canvas)

Amazon SageMaker Canvas (Amazon Web Services, Inc., Seattle, WA, USA) is a managed no-code Machine Learning service that supports data ingestion, preprocessing, model training, and model evaluation [23]. Internally, Canvas relies on AutoGluon [24] to manage the complete training pipeline automatically. For our binary classification task, Canvas was configured to maximize F1 and evaluated ten bagged base model variants (Layer 1): LightGBM, LightGBM Extra Trees, XGBoost, CatBoost, Random Forest (Gini), Random Forest (Entropy), Extra Trees (Gini), Extra Trees (Entropy), FastAI neural network, and PyTorch neural network, each trained on *k*-fold bagged splits of the training data. The final predictor is a two-layer weighted ensemble (Layer 2) built using AutoGluon’s greedy forward ensemble selection algorithm [25]: starting from an empty ensemble, models are added one at a time (with replacement) if each addition improves the validation F1. The weight assigned to each model *k* equals its selection count $n_{k}$ divided by the total ensemble size *S*:

(6) $${\hat{p}}_{\mathrm{ens}}\left(x\right)=\sum_{k=1}^{K}w_{k}\;{\hat{p}}_{k}\left(x\right),\;w_{k}=\frac{n_{k}}{S},$$

where ${\hat{p}}_{k}\left(x\right)$ is the bag-averaged predicted probability from base model *k*, and the final binary decision applies threshold τ from Equation (4). In our workflow, Canvas was also used for quality profiling and feature screening support, as described in Section 3.3.

##### Custom Automatic Machine Learning Workflow (C-AutoML)

We proposed an iterative training methodology where different stratified 80/20 train/validation splits are tested on every model candidate across N=10 outer iterations, yielding 60 total trials (10 iterations × 6 model families). The training core executes an inner loop that iterates through six algorithm families: LR, RF, MLP, XGBoost, LightGBM, and CatBoost.

For Hyperparameter Optimization (HPO), C-AutoML adopts a random search strategy [26]: hyperparameter configuration is drawn uniformly at random from a predefined discrete candidate set using scikit-learn’s ParameterSampler [27] (seed =42+100i+m, where *i* is the outer-loop iteration and *m* is the model-family index). Random search is preferred over grid search because it explores the hyperparameter space more efficiently at a fixed computational budget [26]. The complete search spaces are listed in Table 7.

**Figure 5:**
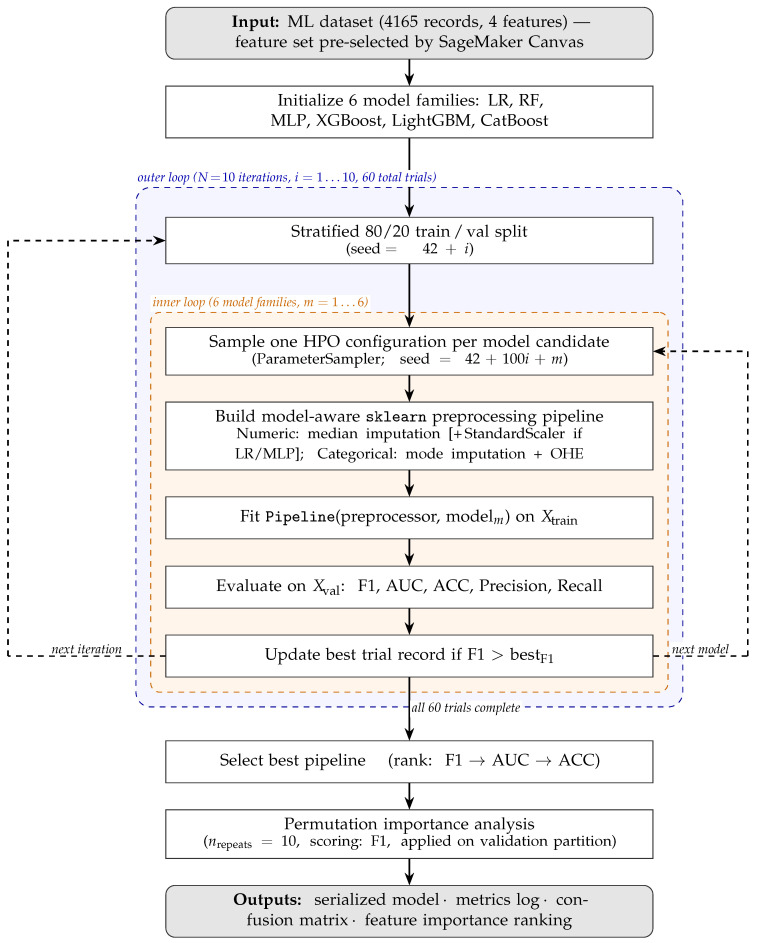
Flowchart of the C-AutoML training pipeline. The outer loop runs N=10 iterations (i=1…10), each drawing a fresh stratified 80/20 train/validation split, for a total of 60 trials (10 iterations × 6 model families). Within each trial, one Hyperparameter Optimization (HPO) configuration is stochastically sampled from a predefined search space (ParameterSampler) and a model-aware sklearn pipeline is fitted on the training partition (median imputation; StandardScaler for LR/MLP; OneHotEncoder for categorical variables). Candidates are ranked by F1-score (primary), AUC (secondary), and ACC (tertiary) across all 60 trials; the best pipeline is then selected and subjected to permutation importance analysis ($n_{\mathrm{repeats}}=10$, F1 scoring) to rank feature contributions.

The proposed methodology fits full scikit-learn (1.6.1) [27] pipelines with model-aware preprocessing (StandardScaler enabled for LR and MLP; disabled for tree-based models) and handles missing values using median imputation for numeric features and mode imputation for categorical features. Model selection is obtained by ranking all 60 trials w.r.t. F1 (primary metric, selected to account for class imbalance), followed by AUC and overall Accuracy (ACC) as tie-breaking metrics. Upon identifying the optimal pipeline, the system performs permutation-based feature importance analysis ($n_{\mathrm{repeats}}=10$, F1 scoring on the validation partition) to enhance model interpretability. Figure 5 provides a flowchart overview of the complete C-AutoML pipeline.

#### 3.6.3. Evaluation Metrics

Performance was evaluated with four complementary metrics: F1, overall ACC, AUC, and confusion matrix analysis. In the cloud-managed workflow (Amazon SageMaker Canvas), the target optimization metric was F1. For our proposed C-AutoML pipeline, F1 was also the primary model-selection criterion because it balances precision and recall under class imbalance; ties in F1 were resolved using AUC and then overall ACC:

(7) $$F1=2\times \frac{\mathrm{Precision}\times \mathrm{Recall}}{\mathrm{Precision}+\mathrm{Recall}}.$$

Overall ACC was reported as the proportion of correctly classified cases among all evaluated pregnancies:

(8) $$\mathrm{Accuracy}=\frac{TP+TN}{TP+TN+FP+FN}.$$

Discrimination across thresholds was quantified with AUC, where higher values indicate better separation between PE and non-PE classes [28].

Finally, the confusion matrix (TP,TN,FP,FN) was analyzed to characterize the clinical error profile of each candidate model, especially the trade-off between missed-risk cases and false alerts [29,30].

## 4. Results

Our results will address the performance of the models (i) with the provided data from the Isidro Ayora Gynecology and Obstetrics Hospital (HGOIA) and (ii) with the synthetic clinical data that we produced. For the former, we trained our models using Amazon SageMaker Canvas and our proposed methodology C-AutoML. For the latter, we only used C-AutoML.

### 4.1. Performance of C-AutoML on HGOIA Data

**Figure 6:**
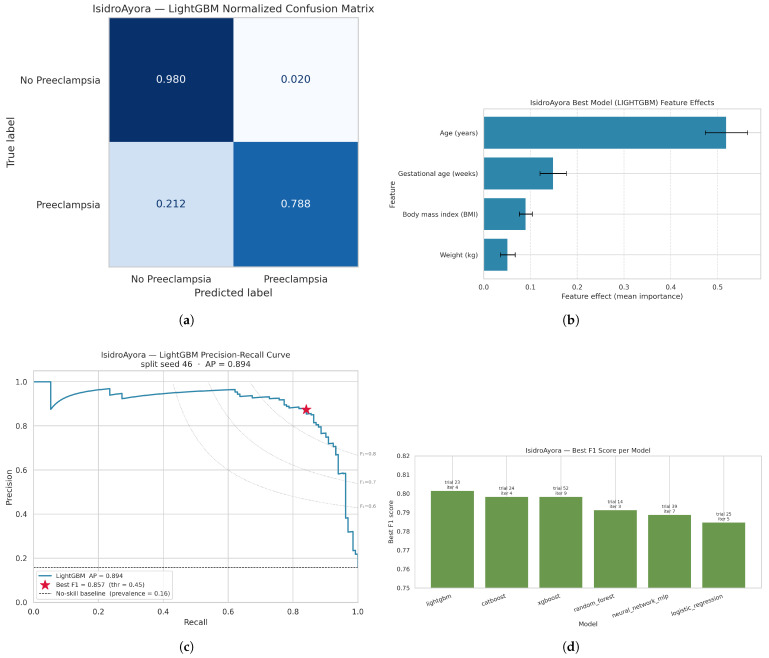
Performance analysis of the best C-AutoML model (LightGBM, Trial 23) on the HGOIA dataset. (**a**) Normalized confusion matrix. (**b**) Feature importance (mean ± std). (**c**) Precision–recall curve. (**d**) F1-score comparison across the best model from each family.

Our proposed methodology C-AutoML achieved its best performance on model LightGBM, with an F1=0.802, Precision=0.837, Recall=0.768, and AUC=0.944, on Trial#23, iteration#4. Table 8 presents the complete performance of the different trials for each of the proposed models. Each iteration uses a different train and validation split and different hyperparameters. Figure 6 presents the normalized confusion matrix (Figure 6a), the feature importance values (Figure 6b), the precision–recall curve performance (Figure 6c), and a comparison w.r.t. the F1 of the models trained in Figure 6d.

### 4.2. Performance of Weighted Ensemble Model on HGOIA Data

Table 9 summarizes the performance of the different models evaluated in Amazon SageMaker Canvas. The best performance was achieved by the Weighted Ensemble Model. Inspection of the downloaded model artifact confirmed that the ensemble comprised three base models selected with equal weights ($w_{k}=\frac{1}{3}$, Equation (6)): LightGBM, Random Forest (Entropy), and NeuralNetFastAI; the remaining seven Layer 1 candidates did not improve validation F1 and were excluded. The ensemble achieved F1=0.805, Precision=0.770, Recall=0.844, and AUC=0.956. Compared to the LightGBM model trained with our proposed C-AutoML, the AWS model improves on the recall performance metric, reducing the number of false negatives. However, the precision of the LightGBM model implies a smaller false-positive rate, which translates into fewer examinations.

**Figure 7:**
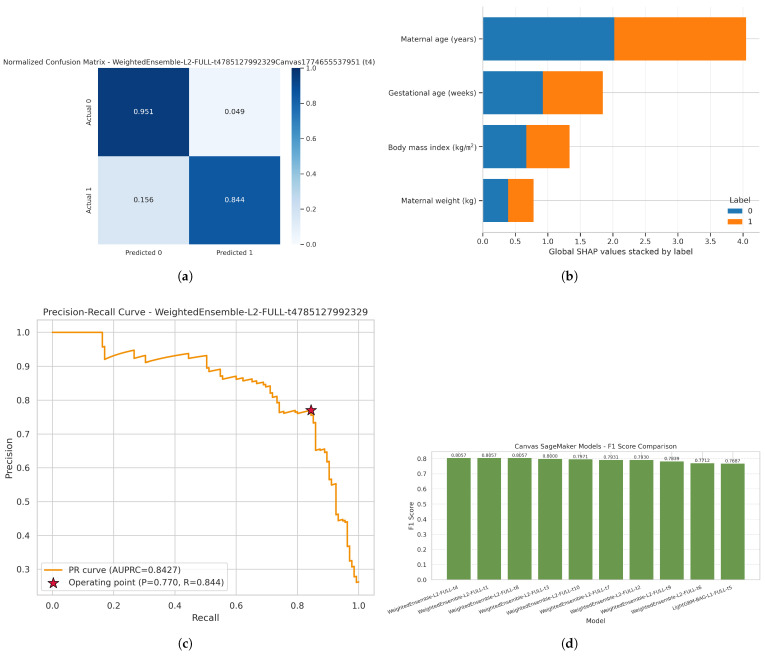
Performance analysis of the Amazon SageMaker Canvas Weighted Ensemble on the HGOIA dataset. (**a**) Normalized confusion matrix. (**b**) KernelSHAP feature importance values for the Amazon SageMaker Weighted Ensemble. (**c**) Precision–recall curve for the Amazon SageMaker Weighted Ensemble model. (**d**) F1-score comparison across all models trained in Amazon SageMaker Canvas.

Figure 7 presents the normalized confusion matrix (Figure 7a), the KernelSHAP values for model interpretability (Figure 7b), the precision–recall curve performance (Figure 7c), and a comparison w.r.t. the F1 of the trained models in AWS SageMaker Canvas in (Figure 7d).

### 4.3. Logistic Regression Model on Clinical Synthetic Generated Data

In this case, our proposed methodology C-AutoML achieved its best performance for model LR, with a perfect score of F1=1.000 on Trial#1, iteration#1. Table 10 presents the complete performance of the different trials for each of the proposed models and iterations. It should be noted that more than one model achieves a similar performance score as LR for the synthetic data. This is structurally expected: the diagnostic label ${\mathrm{PE}}_{\mathrm{MSP}}$ is assigned by the deterministic threshold rule in Equation (5), which applies linear inequality conditions to the same features used as model inputs, producing linearly separable class boundaries that any of the evaluated classifiers can recover. The consistently near-perfect scores therefore confirm that the parametric distributions in Table 6 are sufficiently separated with respect to the MSP rule, validating the generation process itself.

**Figure 8:**
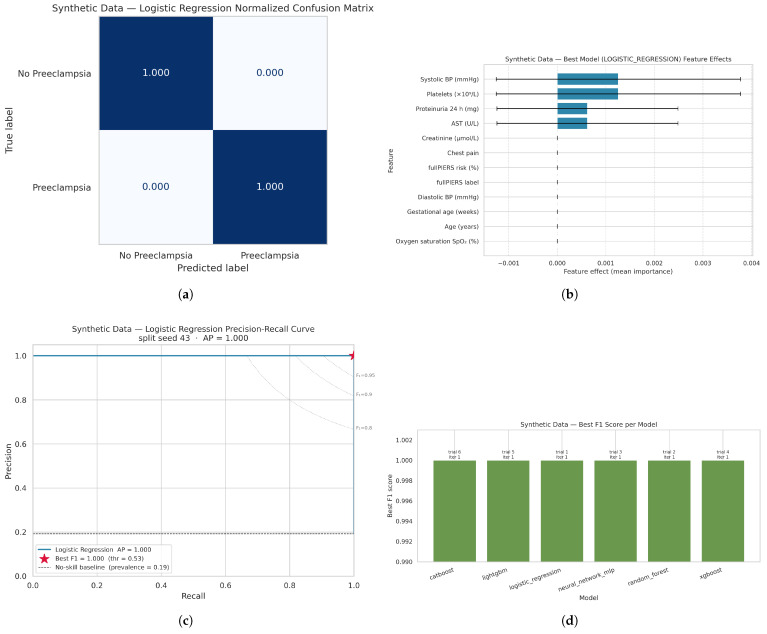
Performance analysis of the best C-AutoML model (Logistic Regression, Trial 1) on the synthetic clinical dataset. (**a**) Normalized confusion matrix. (**b**) Mean and standard deviation of feature importance in the Logistic Regression prediction on synthetic clinical data. (**c**) Precision–recall curve for the best Logistic Regression model on synthetic clinical data. (**d**) F1-score comparison across best model per family on synthetic clinical data.

Figure 8 presents the performance of the LR model according with the normalized confusion matrix (Figure 8a), the feature importance values (Figure 8b), the precision–recall curve (Figure 8c), and a comparison of the F1 among the trained models (Figure 8d). The feature-effects plot indicates that systolic blood pressure, platelet count, 24 h proteinuria, and AST were the dominant contributors in the selected LR model, which is coherent with the clinical rule used to generate the synthetic labels.

### 4.4. Artificial Intelligence Explainability

SHAP, introduced in [31], computes Shapley values to attribute feature importance for individual predictions. In this section, we present feature importance results from three analyses: (i) applying SHAP to the LightGBM model trained on HGOIA data, (ii) evaluating built-in feature importance from Amazon SageMaker Canvas, and (iii) applying SHAP to the best-performing model on synthetic clinical data.

#### SHAP Explainability of LightGBM on HGOIA Dataset

**Figure 9:**
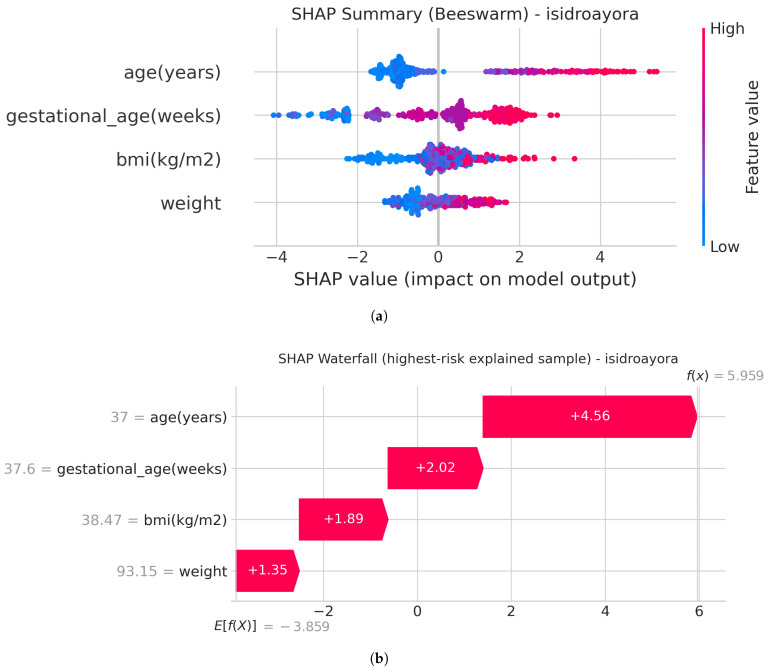
SHAP explainability visualizations for the best LightGBM model trained on the HGOIA dataset. (**a**) Global feature importance via beeswarm plot. (**b**) Local instance-level explanation via waterfall plot for the highest-risk predicted case. (**a**) SHAP beeswarm plot summarizing feature importance and impact direction. Each point represents a SHAP value for a single feature and instance. (**b**) SHAP waterfall plot for a high-risk instance, illustrating how each feature contributes to shifting the model prediction from the base value to the final output.

Figure 9 presents a SHAP beeswarm (Figure 9a) and a SHAP (Figure 9b) waterfall plots that show the feature importance w.r.t. the model output. For LightGBM, it is noticeable that variable Age was the most influential variable, with higher values producing predominantly positive SHAP contributions and thus increasing the predicted probability of PE. GA also showed a strong contribution, with higher values generally associated with positive model output, whereas lower GA tended to reduce predicted risk. In contrast, BMI and weight had more moderate effects centered closer to zero, suggesting that they refined the prediction but contributed less than Age and GA to the final classification. Overall, the explainability analysis supports that the LightGBM model captured demographically plausible risk gradients while remaining consistent with the ranking observed in the performance analysis.

### 4.5. SHAP Explainability of the Weighted Ensemble Model on HGOIA Dataset

**Figure 10:**
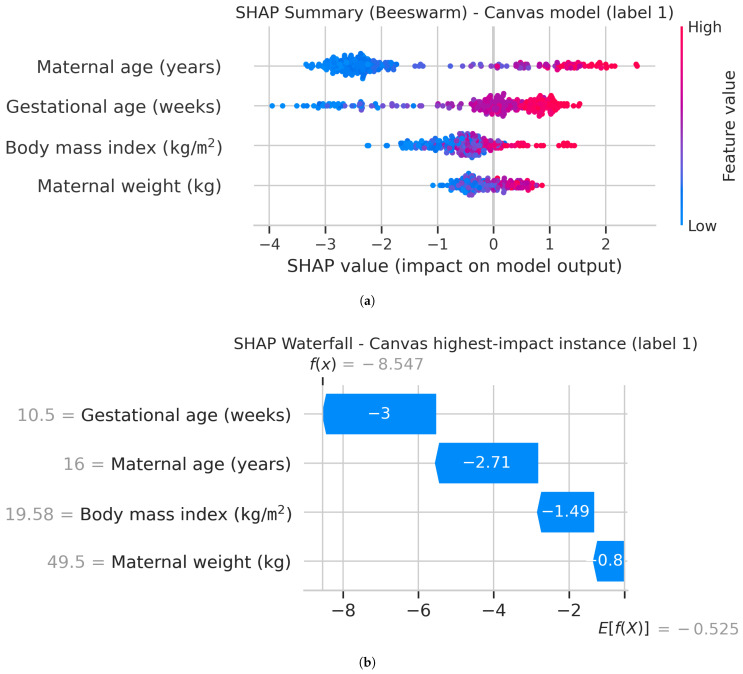
SHAP explainability visualizations for the Amazon SageMaker Canvas Weighted Ensemble Model on the HGOIA dataset. (**a**) Global feature importance via beeswarm plot; maternal age is the dominant contributor (mean |SHAP|=4.046). (**b**) Local instance-level explanation via waterfall plot for the highest-impact predicted case. (**a**) SHAP beeswarm summary plot for the Weighted Ensemble Model. Each point represents a SHAP value for a single feature and instance; color encodes feature value. (**b**) SHAP waterfall plot for the highest-impact instance (index 36), showing how each feature shifts the prediction from the base value E[f(X)]=−0.525 to the final output f(x)=−8.547.

Figure 10 presents the SHAP analysis for the Weighted Ensemble Model trained in Amazon SageMaker Canvas on the HGOIA dataset, including a beeswarm summary plot (Figure 10a) and a waterfall plot for the highest-impact instance (Figure 10b).

The beeswarm plot reveals that maternal age (years) is the dominant driver of model output (mean |SHAP|=4.046), followed by GA (weeks) (mean |SHAP|=1.847) and BMI (mean |SHAP|=1.335). Maternal weight contributes with a smaller but still observable effect. This ranking is consistent with the feature importance observed in the C-AutoML LightGBM model, confirming that maternal age and GA are the most informative demographic predictors in this dataset. Notably, approximately 34.9% of feature contributions are positive and 65.1% are negative across all feature–instance pairs, indicating that feature effects are bidirectional and patient-specific rather than uniformly monotonic. Higher values of maternal age (shown in red) are associated with predominantly positive SHAP contributions, pushing predictions toward PE, while lower values (blue) tend to reduce predicted risk.

The waterfall plot for the highest-impact instance (index 36, f(x)=−8.547, E[f(X)]=−0.525) illustrates a case where all four features act in the same direction. GA at 10.5 weeks contributes −3.002 SHAP units, maternal age at 16 years contributes −2.710, BMI at 19.58 kg/m^2^ contributes −1.486, and maternal weight at 49.5 kg contributes −0.8, cumulatively shifting the prediction well below the base value. This pattern is clinically coherent: a very young patient at early GA with low BMI and weight presents a low-risk demographic profile, and the model correctly assigns a strong negative prediction. Taken together, the global and local SHAP analyses confirm that the Weighted Ensemble Model captures clinically plausible risk gradients from demographic variables alone, and that its decision structure is interpretable and consistent with the known epidemiology of PE.

#### SHAP Explainability of Logistic Regression on Synthetic Clinical Data

**Figure 11:**
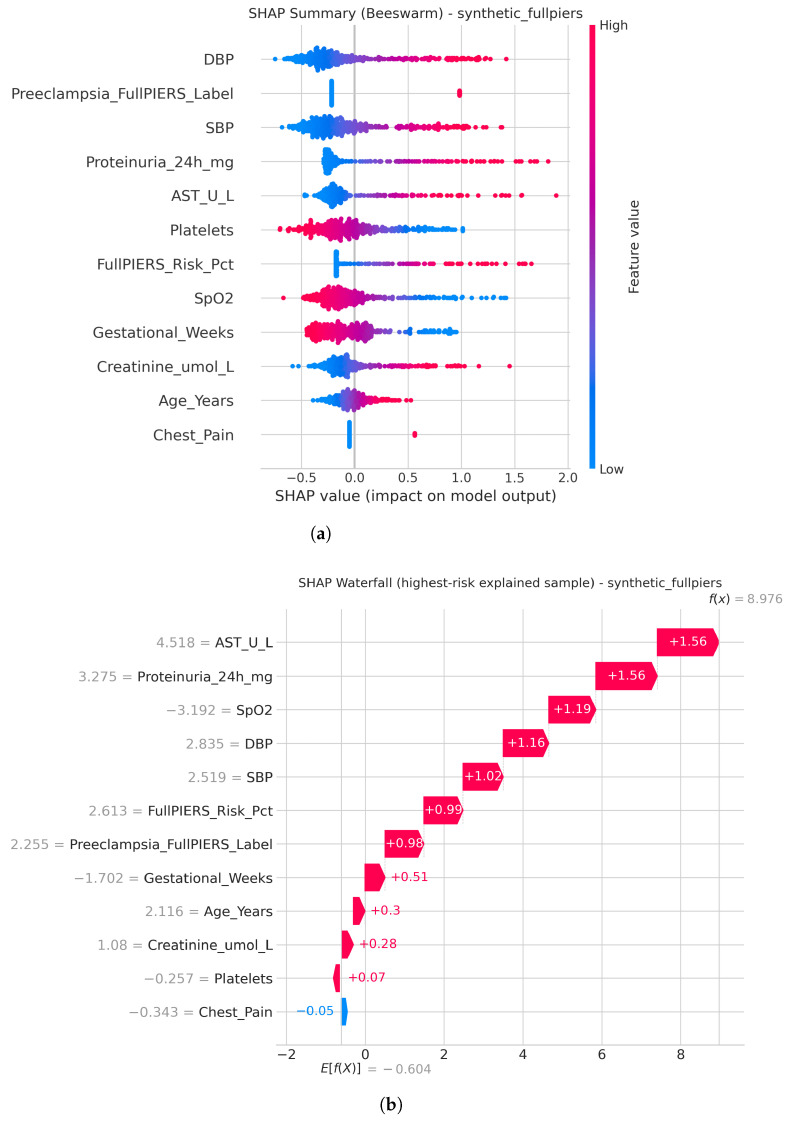
SHAP explainability visualizations for the best LR model trained on the synthetic clinical dataset. (**a**) Global feature importance via beeswarm plot; DBP and SBP are the dominant contributors. (**b**) Local instance-level explanation via waterfall plot for the highest-risk predicted case, dominated by AST, proteinuria, and blood pressure. (**a**) SHAP beeswarm summary for the LR model on synthetic clinical data. Each point represents a SHAP value for a single feature and instance; color encodes feature value. (**b**) SHAP waterfall plot for the highest-risk instance (f(x)=8.976, E[f(X)]=−0.604), showing cumulative feature contributions from the base value to the final output.

Figure 11 presents the SHAP analysis for the LR model trained on the synthetic clinical dataset, including a beeswarm summary plot (Figure 11a) and a waterfall plot for the highest-risk instance (Figure 11b).

The beeswarm plot reveals a feature-importance structure that is highly consistent with the rule-based and prognostic variables used to construct the data. DBP and SBP appear among the strongest positive contributors, with higher values (red) shifting the prediction toward PE. Proteinuria_24h_mg, AST, creatinine, and the FullPIERS risk percentage also display predominantly positive SHAP values at higher feature levels, indicating that the model assigns greater risk when these markers of renal, hepatic, and overall maternal severity increase. In contrast, higher platelet counts, ${\mathrm{SpO}}_{2}$, and GA tend to move predictions toward lower risk, reflected by negative SHAP contributions at higher values. Age shows a smaller but still coherent contribution, while chest pain has a limited overall effect due to its low prevalence in the dataset.

The waterfall plot for the highest-risk instance (f(x)=8.976, E[f(X)]=−0.604) confirms this interpretation at the local level. AST (=4.518 U/L) and Proteinuria_24h_mg (=3.275 mg) each contribute +1.56 SHAP units, followed by low ${\mathrm{SpO}}_{2}$ (=−3.192) (feature values shown in the waterfall plot correspond to the scaled representations used internally by the LR pipeline in C-AutoML) at +1.19, DBP (=2.835) at +1.16, and SBP (=2.519) at +1.02. The FullPIERS risk percentage and the FullPIERS diagnostic label add a further +0.99 and +0.98 respectively, while chest pain makes a small negative contribution of −0.05. The additive accumulation of hemodynamic, renal, and hepatic abnormalities drives the final output far above the base value, confirming that the LR model identifies high-risk cases through a clinically coherent combination of severity indicators. Taken together, the global and local SHAP analyses demonstrate that the model learned a decision structure dominated by classical PE risk markers and is fully consistent with the rule-based labels used during synthetic data generation.

## 5. Discussion

In this study, we explored different models and approaches to train a robust model for the prediction of PE. To this end, we used the data provided by the Isidro Ayora Gynecology and Obstetrics Hospital (HGOIA). However, the digital records are predominantly demographic. For this reason, we generated a synthetic dataset using variables commonly found in PIERS.

For the HGOIA dataset, we trained models with our proposed methodology named C-AutoML, where we compared the performance of up to six different Machine Learning models. Our approach aims to improve performance by exposing the models to different training and validation splits during each trial and also searches for the best hyperparameter configuration. The best model found corresponded to LightGBM. In parallel, we trained additional Machine Learning models using Amazon SageMaker Canvas, which produced as best model a Weighted Ensemble Model. Finally, for the clinical synthetic dataset, LR, among other models, achieved ideal performance.

Our results show that the Weighted Ensemble Model achieved the best performance for the HGOIA dataset because it has the highest recall (Recall=0.844), compared to that of LightGBM (Recall=0.768). This is important because recall (sensitivity) measures the proportion of women with PE that the model correctly identified. For a medical setting, it is necessary that the model has a high recall because that lowers the false negatives. As a matter of fact, the Weighted Ensemble Model loses one in six cases of PE. On the other hand, the LightGBM trained with our proposed C-AutoML approach has a higher precision (Precision=0.837) compared to the Weighted Ensemble Model (Precision=0.770). This result is advantageous because higher precision implies fewer false alarms. Consequently, it is recommended to combine both of these models for prediction at the health facility.

For the synthetic dataset, all six evaluated model families achieve near-perfect performance (F1≥0.988), with LR, RF, MLP, XGBoost, LightGBM, and CatBoost all reaching F1=1.000 in the first three iterations. This high performance is a design consequence: diagnostic labels are assigned by a deterministic rule (Equation (5)) applied to the same features used as model inputs, creating linearly separable class boundaries. The synthetic track serves as a controlled feasibility experiment that confirms the C-AutoML pipeline operates correctly on data with a known ground truth to predict PE; it does not constitute a clinical performance claim. Notably, the inclusion of ${\mathrm{SpO}}_{2}$, chest pain, platelet count, creatinine, and AST in the synthetic track is grounded in the published FullPIERS equation (Equation (2)), which uses precisely these variables as predictors [9]. However, it must be noticed that the purpose of  (2) is to predict adverse outcomes of PE, which differs from our main purpose.

The interpretability analysis shows the feature importance of the variables of each dataset for prediction. For the HGOIA dataset, the most important features are: patient’s age, GA, and BMI. On the other hand, for the generated synthetic data, systolic blood pressure, platelet count and 24 h proteinuria lead the impact on model’s prediction.

### 5.1. Comparison with Similar Frameworks

Table 11 situates our results among studies that employ comparable model families and predictor types. Despite using only four demographic variables, our best models achieve discrimination metrics (AUC 0.944–0.956, F1 0.802–0.805) that fall within the range reported for tree-based ensembles operating on richer predictor sets [5,32,33,34]. Direct numerical ranking across studies is discouraged by the substantial heterogeneity in populations, predictor availability, and outcome definitions documented in recent systematic reviews [7,8,35]. Consequently, the comparison in Table 11 is intended to contextualize rather than rank performance.

### 5.2. Limitations

Several limitations of this study should be considered. First, the HGOIA dataset contains only demographic variables (age, weight, BMI, and GA in weeks); the absence of laboratory and clinical measurements restricts predictive granularity and precludes direct comparison with clinically richer models. Second, this is a single-center, retrospective study conducted at a single public hospital; generalizability to other institutions or geographic contexts remains to be evaluated. Third, the train–validation split in C-AutoML was performed at the record level rather than the patient level. Given that the source dataset contains on average 1.81 records per patient (range 1–13), the reported validation metrics may be modestly optimistic and patient-level cross-validation is recommended for future work. Finally, the synthetic dataset, while useful for assessing the feasibility of our proposed framework, does not constitute external clinical validation.

Regarding patient selection, the positive class was constructed exclusively from records matching ICD-10 codes O11, O13, O14, O15, and O16, as documented in Table 1. Patients with pre-existing chronic hypertension without superimposed preeclampsia (ICD-10 O10) were absent from both classes by design: the negative class consists solely of normal pregnancy supervision records (ICD-10 Z340, Z348, Z349). However, cases of superimposed preeclampsia on chronic hypertension (ICD-10 O11) are present in the positive class and represent a subgroup with a physiopathological background that partially differs from de novo gestational preeclampsia; separate analysis of O11 cases is recommended as future work. Additionally, stratified analysis by PE sub-category (early vs. late onset; moderate vs. severe) was not feasible with the available data, which records binary PE diagnosis without severity grade or GA at onset; subgroup analysis constitutes a direction for future research.

Notwithstanding these limitations, this study benefits from a real-world hospital dataset, a reproducible C-AutoML pipeline with full artifact tracking via MLflow, SHAP-based interpretability, and benchmarking against a commercial AutoML platform (Amazon SageMaker Canvas).

## 6. Conclusions

This study explored the performance of different Machine Learning models towards the prediction of PE in the population of women at the Isidro Ayora Gynecology and Obstetrics Hospital (HGOIA). Amazon SageMaker Canvas was pivotal in assessing the predictive power of each of the features from the provided dataset. Selecting features with high predictive power prevented our models from overfitting, improving training. To the best of our knowledge, this is the first study combining patient datasets from Ecuador with Machine Learning. Our results show that a combination of our proposed methodology with the trained model from Canvas can lead to effective predictions at the health facility.

Our proposed training methodology improved the hyperparameter settings of different models targeting the F1 metric, which balances precision and recall, where the latter is of most importance for a medical application because it measures the proportion of true PE cases correctly identified by the model, whereas the former accounts for the cost of false alarms. We prioritize recall because it emphasizes minimizing missed diagnoses.

LightGBM achieved the best performance w.r.t. the F1 metric using our proposed C-AutoML workflow, with F1=0.802, Recall=0.768, and Precision=0.837. This means that 77% of true PE cases were correctly detected by the model. Consequently, this model misses one out of four true PE cases. On the other hand, the Weighted Ensemble Model trained in Amazon SageMaker Canvas achieved an F1=0.805, Precision=0.770, and Recall=0.844. Therefore, 84% of true PE cases were correctly detected by the ensemble model and misses 1 out of 6 true PE cases. This improvement over our single model is due to the ensemble model. We suggest a combination of both models to aid physicians in their decision-making when diagnosing PE, given that the Weighted Ensemble Model provides a recall greater than 0.80, which can be considered clinically reasonable if used alongside clinical judgment, and that LightGBM achieves an improved precision that can reduce false positives and improve resource utilization.

We look forward to improving our results in the near future, if additional data is provided with more clinical information. Hence, this initial work can be extended to obtain a PIERS model that can support health facilities in Ecuador. It should be noted that our models trained on the dataset from HGOIA use a few demographic variables. This could support preventive diagnosis in settings where health facilities are scarce or expensive.

The proposed methodology can be considered an initial framework adaptable to other health problems in the Ecuadorian clinical reality with minimal reconfiguration. It addresses class imbalance, which is a common challenge in medical datasets, and incorporates SHAP-based model interpretability to support physician decision-making. Additionally, model benchmarking and the integration of Machine Learning Operation (MLOP) practices provide an active Machine Learning cycle, enabling continuous improvement as new data become available.

## Figures and Tables

**Table 1:** ICD-10 codes used to define the binary target variable for PE classification. The positive class (PE = 1) indicates the presence of the disorder, while the negative class (PE = 0) corresponds to normal pregnancy supervision codes.

Preeclampsia	ICD-10	ICD-10 Description	Total Cases
0	Z340	Supervision of normal first pregnancy	2951
0	Z348	Supervision of other normal pregnancies	537
0	Z349	Supervision of normal pregnancy, unspecified	83
1	O149	Preeclampsia, unspecified	221
1	O140	Mild to moderate preeclampsia	209
1	O13X	Gestational hypertension [pregnancy-induced]	180
1	O141	Severe preeclampsia	36
1	O11X	Pre-existing hypertension with superimposed preeclampsia	26
1	O142	HELLP syndrome	1
1	O16X	Maternal hypertension, unspecified	1

**Table 2:** Summary of Amazon SageMaker Canvas data-quality report before final filtering.

Metric	Value
Number of features	23
Number of rows	4245
Missing values	13.6%
Valid values	86.4%
Duplicate rows	1.84%
Numeric features	12
Categorical features	7
Text features	0
Datetime features	1
Binary features	2
Unknown type features	0

**Table 3:** Summary of dataset characteristics for the HGOIA preeclampsia classification dataset. Each record corresponds to a prenatal clinical visit; a single patient may contribute multiple records across visits.

Characteristic	Value
Data collection period	2022–2025
Total records after ICD-10 filter and overlap removal	4245
Unique patients (source dataset)	2344
Average records per patient	1.81 (range: 1–13)
Duplicate/low-signal records removed (Canvas)	80 (1.88%)
Final ML dataset—total records	4165
No preeclampsia—class 0	3494 (83.89%)
Preeclampsia—class 1	671 (16.11%)
Class imbalance ratio (class 0:class 1)	5.2:1
Initial column-level missingness (pre-cleaning)	12.9%
Missing data handling	Columns with high missingness removed; no imputation applied
Missing values in final dataset	0
Predictor features	4 (age, weight, BMI, gestational weeks)
Train/validation split (C-AutoML)	80%/20%, record-level, stratified by class

**Table 4:** Descriptive statistics of demographic variables by preeclampsia diagnosis in pregnant women at HGOIA from years 2022 to 2025. Statistics are computed on the pre-deduplication cohort (4245 records: 3571 normal, 674 preeclampsia); the final ML dataset after Canvas processing contains 4165 records (see Table 3).

Variable	Preeclampsia	Sample Size	Mean	Median	Std. Dev.	*p*-Value ^a^
AGE (YEARS)	No	3571	18.71	18.00	4.13	<0.001
	Yes	674	29.72	30.00	7.17	
BMI	No	3571	25.71	25.19	4.39	<0.001
	Yes	674	33.00	32.63	6.02	
GESTATIONAL WEEKS	No	3571	27.55	27.70	7.76	<0.001
	Yes	674	33.80	35.40	4.42	
WEIGHT	No	3571	61.20	59.50	11.67	<0.001
	Yes	674	80.51	78.75	16.17	

^a^ Mann–Whitney *U* test, two-sided; compares each variable between the preeclampsia and normal groups.

**Table 5:** Distribution of pregnancies and preeclampsia prevalence across demographic concentration quartiles in women at HGOIA from years 2022 to 2025.

Variable	Bin (Range)	Total Pregnancies	Normal Pregnancies	Preeclampsia Pregnancies	Preeclampsia Prevalence (%)	*p*-Value ^b^
AGE (YEARS)	(12.999, 17.0]	1745	1704	41	2.35%	<0.001
	(17.0, 18.0]	690	670	20	2.90%	
	(18.0, 22.0]	797	735	62	7.78%	
	(22.0, 48.0]	1013	462	551	54.39%	
AGE GROUP	10–14 years	73	66	7	9.59%	<0.001
	15–19 years	2877	2813	64	2.22%	
	20–35 years	1100	663	437	39.73%	
	36–49 years	195	29	166	85.13%	
BMI	(15.329, 23.18]	1063	1041	22	2.07%	<0.001
	(23.18, 25.93]	1064	1014	50	4.70%	
	(25.93, 29.42]	1058	935	123	11.63%	
	(29.42, 73.46]	1060	581	479	45.19%	
GESTATIONAL WEEKS	(3.399, 26.0]	1066	1055	11	1.03%	<0.001
	(26.0, 27.696]	1229	1062	167	13.59%	
	(27.696, 35.2]	904	760	144	15.93%	
	(35.2, 41.5]	1046	694	352	33.65%	
WEIGHT	(19.799, 54.0]	1086	1065	21	1.93%	<0.001
	(54.0, 61.5]	1043	1002	41	3.93%	
	(61.5, 71.5]	1064	937	127	11.94%	
	(71.5, 157.2]	1052	567	485	46.10%	

^b^ Pearson chi-squared test of independence between variable bin and preeclampsia status.

**Table 6:** Statistical distributions for synthetic data generation.

Feature	Control Group	Risk Group	Units
Age	N(27,4)	N(33,6)	years
Gestational age	U(34,40)	U(26,37)	weeks
Systolic BP	N(115,8)	N(155,12)	mmHg
Diastolic BP	N(75,5)	N(102,10)	mmHg
Proteinuria	Gamma(2,40)	N(1500,700)	mg/24 h
${\mathrm{SpO}}_{2}$	N(98,1)	N(93,3)	%
Platelets	N(240,40)	N(110,55)	$10^{9}$/L
Creatinine	N(60,10)	N(110,40)	μmol/L
AST	N(25,6)	N(90,50)	U/L
Chest pain	Bernoulli(0.03)	Bernoulli(0.35)	binary

Notes: N(μ,σ): Normal distribution with mean μ and standard deviation σ; U(a,b): Uniform distribution over the interval [a,b]; Gamma(k,θ): Gamma distribution with shape parameter *k* and scale parameter θ; Bernoulli(p): Bernoulli variable with success probability *p* (binary outcome: 0 = absent, 1 = present).

**Table 7:** C-AutoML hyperparameter search spaces. At each trial one configuration is drawn uniformly at random (ParameterSampler) from the Cartesian product of the listed candidate values. Tree-based models omit StandardScaler; LR and MLP apply it.

Model Family	Hyperparameter	Candidate Values
Logistic Regression	Regularization *C*	{0.01, 0.1, 1, 5, 10}
	Solver	{liblinear, lbfgs}
	Class weight	{none, balanced}
Random Forest	No. of estimators	{200, 400, 600}
	Maximum depth	{none, 5, 10, 20}
	Min. samples per split	{2, 5, 10}
	Min. samples per leaf	{1, 2, 4}
	Class weight	{none, balanced, balanced_subsample}
MLP Neural Network	Hidden-layer sizes	{(64), (128), (64, 32), (128, 64)}
	L2 penalty α	{0.0001, 0.001, 0.01}
	Initial learning rate	{0.0005, 0.001, 0.005}
XGBoost	Maximum tree depth	{3, 4, 6, 8}
	Learning rate	{0.01, 0.05, 0.1, 0.2}
	Subsample ratio	{0.70, 0.85, 1.0}
	Column sample by tree	{0.70, 0.85, 1.0}
	L2 regularization λ	{1, 2, 5}
LightGBM	Maximum leaf count	{15, 31, 63}
	Learning rate	{0.01, 0.05, 0.1}
	Subsample ratio	{0.70, 0.85, 1.0}
	Column sample by tree	{0.70, 0.85, 1.0}
	L2 regularization λ	{0, 1, 5}
CatBoost	Tree depth	{4, 6, 8}
	Learning rate	{0.01, 0.05, 0.1}
	Number of boosting rounds	{200, 400, 600}
	L2 leaf regularization	{1, 3, 5}

**Table 8:** C-AutoML performance across trials and iterations on the HGOIA dataset. Each trial uses a different train/validation split and hyperparameter configuration.

Trial	Iter	Model	F1	Acc	Prec	Rec	AUC
1	1	LR	0.7344	0.9184	0.7705	0.7015	0.9318
2	1	RF	0.7368	0.9220	0.8053	0.6791	0.9341
3	1	MLP	0.7410	0.9220	0.7949	0.6940	0.9044
4	1	XGBoost	0.7431	0.9220	0.7899	0.7015	0.9384
5	1	LightGBM	0.7120	0.9136	0.7672	0.6642	0.9274
6	1	CatBoost	0.7460	0.9232	0.7966	0.7015	0.9393
7	2	LR	0.7417	0.9256	0.8396	0.6642	0.9467
8	2	RF	0.7713	0.9196	0.7107	0.8433	0.9506
9	2	MLP	0.7739	0.9292	0.7953	0.7537	0.9381
10	2	XGBoost	0.7786	0.9304	0.7969	0.7612	0.9558
11	2	LightGBM	0.7790	0.9292	0.7820	0.7761	0.9535
12	2	CatBoost	0.7510	0.9244	0.7983	0.7090	0.9525
13	3	LR	0.7296	0.9004	0.6474	0.8358	0.9482
14	3	RF	0.7914	0.9304	0.7639	0.8209	0.9421
15	3	MLP	0.7405	0.9184	0.7578	0.7239	0.9315
16	3	XGBoost	0.7382	0.9268	0.8687	0.6418	0.9478
17	3	LightGBM	0.7667	0.9328	0.8679	0.6866	0.9425
18	3	CatBoost	0.7809	0.9340	0.8376	0.7313	0.9472
19	4	LR	0.7121	0.8884	0.6085	0.8582	0.9428
20	4	RF	0.7557	0.9100	0.6705	0.8657	0.9436
21	4	MLP	0.7871	0.9364	0.8522	0.7313	0.9459
22	4	XGBoost	0.7795	0.9328	0.8250	0.7388	0.9362
23	4	**LightGBM**	**0.8016**	**0.9388**	**0.8374**	**0.7687**	**0.9443**
24	4	CatBoost	0.7984	0.9388	0.8487	0.7537	0.9462
25	5	LR	0.7848	0.9388	0.9029	0.6940	0.9478
26	5	RF	0.7389	0.9016	0.6444	0.8657	0.9512
27	5	MLP	0.7805	0.9352	0.8571	0.7164	0.9443
28	5	XGBoost	0.7938	0.9364	0.8293	0.7612	0.9553
29	5	LightGBM	0.7755	0.9340	0.8559	0.7090	0.9469
30	5	CatBoost	0.7791	0.9340	0.8435	0.7239	0.9453
31	6	LR	0.7101	0.8932	0.6301	0.8134	0.9338
32	6	RF	0.7311	0.9232	0.8365	0.6493	0.9418
33	6	MLP	0.7511	0.9292	0.8641	0.6642	0.9327
34	6	XGBoost	0.7531	0.9292	0.8571	0.6716	0.9457
35	6	LightGBM	0.7542	0.9304	0.8725	0.6642	0.9439
36	6	CatBoost	0.7667	0.9328	0.8679	0.6866	0.9396
37	7	LR	0.7147	0.8860	0.5980	0.8881	0.9457
38	7	RF	0.7758	0.9244	0.7415	0.8134	0.9478
39	7	MLP	0.7888	0.9364	0.8462	0.7388	0.9526
40	7	XGBoost	0.7667	0.9328	0.8679	0.6866	0.9495
41	7	LightGBM	0.7668	0.9292	0.8151	0.7239	0.9506
42	7	CatBoost	0.7843	0.9340	0.8264	0.7463	0.9535
43	8	LR	0.7186	0.9220	0.8557	0.6194	0.9383
44	8	RF	0.7450	0.9088	0.6768	0.8284	0.9418
45	8	MLP	0.7529	0.9244	0.7934	0.7164	0.9352
46	8	XGBoost	0.7339	0.9208	0.7982	0.6791	0.9386
47	8	LightGBM	0.7243	0.9196	0.8073	0.6567	0.9397
48	8	CatBoost	0.7360	0.9208	0.7931	0.6866	0.9296
49	9	LR	0.7107	0.8896	0.6141	0.8433	0.9419
50	9	RF	0.7550	0.9112	0.6786	0.8507	0.9444
51	9	MLP	0.7686	0.9292	0.8099	0.7313	0.9454
52	9	XGBoost	0.7984	0.9388	0.8487	0.7537	0.9448
53	9	LightGBM	0.7922	0.9364	0.8347	0.7537	0.9424
54	9	CatBoost	0.7860	0.9340	0.8211	0.7537	0.9418
55	10	LR	0.6894	0.8800	0.5904	0.8284	0.9349
56	10	RF	0.7666	0.9196	0.7190	0.8209	0.9398
57	10	MLP	0.7317	0.9208	0.8036	0.6716	0.9370
58	10	XGBoost	0.7347	0.9220	0.8108	0.6716	0.9309
59	10	LightGBM	0.7137	0.9172	0.8037	0.6418	0.9359
60	10	CatBoost	0.7540	0.9256	0.8051	0.7090	0.9341

Abbreviations—LR: Logistic Regression; RF: Random Forest; MLP: Multi-Layer Perceptron; XGBoost: Extreme Gradient Boosting; LightGBM: Light Gradient Boosting Machine; CatBoost: Categorical Boosting. **Bold** row indicates the best-performing model overall.

**Table 9:** Performance of models evaluated in Amazon SageMaker Canvas. **Bold** row indicates the default (best) model selected by Canvas.

Model Prefix ^†^	F1 (%)	Acc (%)	AUC	Bal. Acc (%)	Prec (%)	Rec (%)	Log Loss	Latency (s)
FULL-t4785127992329 (default)	**80.565**	**93.405**	**0.956**	**89.790**	**77.027**	**84.444**	**0.210**	**0.162**
FULL-t9785127992329	78.388	92.926	0.954	87.412	77.536	79.259	0.193	0.178
FULL-t8785127992329	80.565	93.405	0.956	89.790	77.027	84.444	0.210	0.184
FULL-t7785127992329	79.310	92.806	0.955	89.731	74.194	85.185	0.225	0.321
FULL-t6785127992329	77.124	91.607	0.957	89.913	69.006	87.407	0.269	0.094
FULL-t5785127992329	80.000	93.165	0.956	89.647	76.000	84.444	0.226	0.099
FULL-t2785127992329	79.298	92.926	0.956	89.205	75.333	83.704	0.211	0.207
FULL-t1785127992329	80.565	93.405	0.956	89.790	77.027	84.444	0.210	0.165
FULL-t10785127992329	79.710	93.285	0.954	88.523	78.014	81.481	0.192	0.152
-t5785127992329	76.873	91.487	0.955	89.841	68.605	87.407	0.264	0.086

^†^ All model names share the common suffix Canvas1774655537951; full name is <prefix>-Canvas1774655537951.

**Table 10:** C-AutoML performance across trials and iterations on the synthetic clinical dataset. Each trial uses a different train/validation split and hyperparameter configuration.

Trial	Iter	Model	F1	Acc	Prec	Rec	AUC
1	1	**LR**	**1.0000**	**1.0000**	**1.0000**	**1.0000**	**1.0000**
2	1	RF	1.0000	1.0000	1.0000	1.0000	1.0000
3	1	MLP	1.0000	1.0000	1.0000	1.0000	1.0000
4	1	XGBoost	1.0000	1.0000	1.0000	1.0000	1.0000
5	1	LightGBM	1.0000	1.0000	1.0000	1.0000	1.0000
6	1	CatBoost	1.0000	1.0000	1.0000	1.0000	1.0000
7	2	LR	1.0000	1.0000	1.0000	1.0000	1.0000
8	2	RF	1.0000	1.0000	1.0000	1.0000	1.0000
9	2	MLP	1.0000	1.0000	1.0000	1.0000	1.0000
10	2	XGBoost	1.0000	1.0000	1.0000	1.0000	1.0000
11	2	LightGBM	1.0000	1.0000	1.0000	1.0000	1.0000
12	2	CatBoost	1.0000	1.0000	1.0000	1.0000	1.0000
13	3	LR	1.0000	1.0000	1.0000	1.0000	1.0000
14	3	RF	1.0000	1.0000	1.0000	1.0000	1.0000
15	3	MLP	1.0000	1.0000	1.0000	1.0000	1.0000
16	3	XGBoost	1.0000	1.0000	1.0000	1.0000	1.0000
17	3	LightGBM	1.0000	1.0000	1.0000	1.0000	1.0000
18	3	CatBoost	1.0000	1.0000	1.0000	1.0000	1.0000
19	4	LR	0.9938	0.9975	0.9877	1.0000	1.0000
20	4	RF	0.9938	0.9975	0.9877	1.0000	0.9998
21	4	MLP	0.9938	0.9975	0.9877	1.0000	1.0000
22	4	XGBoost	0.9938	0.9975	0.9877	1.0000	1.0000
23	4	LightGBM	1.0000	1.0000	1.0000	1.0000	1.0000
24	4	CatBoost	0.9938	0.9975	0.9877	1.0000	1.0000
25	5	LR	0.9875	0.9950	0.9875	0.9875	1.0000
26	5	RF	0.9938	0.9975	0.9877	1.0000	1.0000
27	5	MLP	0.9938	0.9975	0.9877	1.0000	0.9999
28	5	XGBoost	0.9938	0.9975	0.9877	1.0000	1.0000
29	5	LightGBM	1.0000	1.0000	1.0000	1.0000	1.0000
30	5	CatBoost	0.9938	0.9975	0.9877	1.0000	1.0000

Abbreviations—LR: Logistic Regression; RF: Random Forest; MLP: Multi-Layer Perceptron; XGBoost: Extreme Gradient Boosting; LightGBM: Light Gradient Boosting Machine; CatBoost: Categorical Boosting. **Bold** row indicates the best-performing model overall.

**Table 11:** Comparison of selected Machine Learning studies for preeclampsia prediction using tabular demographic and clinical data.

Study	Population/Data Source	Main Predictors	Best Model and Performance
Li et al. (2021) [5]	3759 pregnancies, EHR from a tertiary hospital in China	38 routine clinical parameters at early second trimester (incl. fasting plasma glucose, mean blood pressure, BMI)	XGBoost; AUC 0.955, accuracy 0.920 (F1 0.571)
Liu et al. (2022) [32]	EMR-based cohort with longitudinal pregnancy data	Repeated measures of vital signs and laboratory values across gestation	Ensemble ML on trajectories; AUC >0.90 for late-gestation PE
Zeng et al. (2024) [33]	Population-based Chinese cohort	Maternal characteristics and first-trimester biomarkers (e.g., PLGF, PAPP-A)	Ensemble ML; AUC 0.80–0.86
Advancing ML tools (2025) [34]	Hospital-based cohort	Demographic and clinical variables (incl. blood pressure and laboratory markers)	CatBoost, LightGBM, XGBoost, RF; accuracy 0.90, AUC >0.90
**This study (HGOIA)**	**4165 pregnancies, public hospital in Ecuador**	**Four demographic variables (age, gestational age, BMI, weight)**	**LightGBM (C-AutoML): F1 0.802, AUC 0.944; Weighted Ensemble (Canvas): F1 0.805, AUC 0.956**

**Figure A1:**
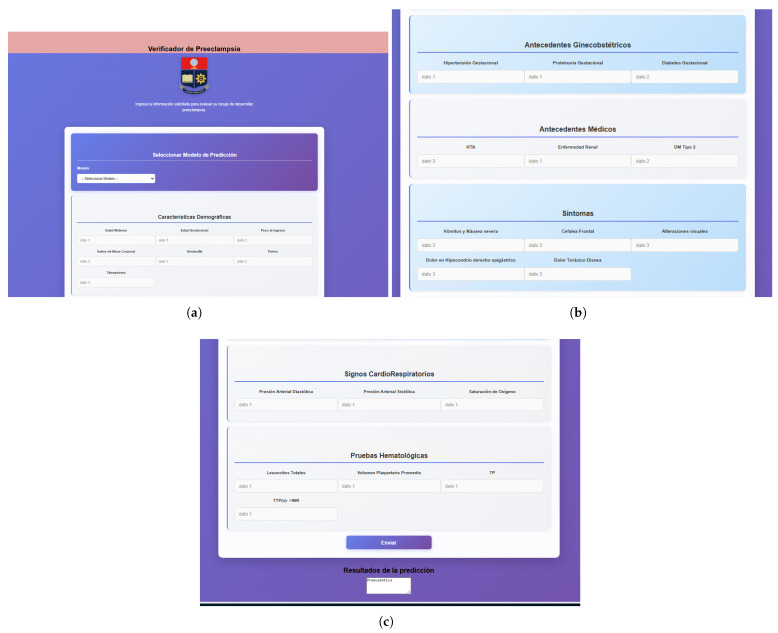
Web interface of the *Verificador de Preeclampsia* system deployed at the Escuela Politécnica Nacional. (**a**) Model selector and demographic input fields. (**b**) Clinical history and symptom input fields. (**c**) Cardiorespiratory and haematological input fields with the prediction results panel. (**a**) Model selection and demographic characteristics section. (**b**) Gyneco-obstetric history, medical history, and symptoms section. (**c**) Cardiorespiratory signs, haematological tests, submission button, and prediction output panel.

## Data Availability

The anonymized patient dataset from the Isidro Ayora Gynecology and Obstetrics Hospital HGOIA used in this study is not publicly available due to institutional data governance restrictions. The code for the C-AutoML pipeline, trained model artefacts, and supplementary figures are available in the project repository at https://github.com/LeninGF/preeclampsiaML accessed on 19 March 2026.

## Abbreviations

The following abbreviations are used in this manuscript:

ACC	Accuracy
API	Application Programming Interface
AST	Aspartate Aminotransferase
AUC	Area Under the Receiver Operating Characteristic Curve
BMI	Body Mass Index
C-AutoML	Custom Automated Machine Learning Workflow
DBP	Diastolic Blood Pressure
EHR	Electronic Health Record
EN	Elastic Net Logistic Regression
EPN	Escuela Politécnica Nacional
F1	F1-score
GA	Gestational Age
HDP	Hypertensive Disorders of Pregnancy
HGOIA	Isidro Ayora Gynecology and Obstetrics Hospital
HPO	Hyperparameter Optimization
HTN	Hypertension
ICD-10	International Classification of Diseases
LR	Logistic Regression
ML	Machine Learning
MLP	Multi-Layer Perceptron
MLOPs	Machine Learning Operations
MSP	Ministerio de Salud Pública
PE	Preeclampsia
PIERS	Preeclampsia Integrated Estimate of RiSk
PUCE	Pontifical Catholic University of Ecuador
RF	Random Forest
SBP	Systolic Blood Pressure
SHAP	SHapley Additive exPlanations
SVM	Support Vector Machine
ULN	Upper Limit of Normal
WHO	World Health Organization
w.r.t.	with respect to

## Appendix A. Custom Automated Machine Learning Workflow (C-AutoML)

The proposed Custom Automated Machine Learning Workflow (C-AutoML) model selection and hyperparameter optimization algorithm, presented in Algorithm A1, is designed to systematically explore multiple model families and hyperparameter configurations for the binary classification of PE patients. The following sections describe the key components of the algorithm in detail.

### Appendix A.1. Input Processing and Data Preparation

Given a CSV dataset D and a target column name *t*, the algorithm first resolves the exact column name through case-insensitive matching. A stratified train–validation split is performed using the train_test_split function from scikit-learn [27], parameterized by the test proportion τ and a seed-dependent random state $s_{\mathrm{split}}=s_{\mathrm{base}}+i$, where *i* denotes the current search iteration.

The binary target variable y is encoded using LabelEncoder, producing integer labels {0,1}. A mapping M:{classname↦integer} is preserved for interpretability.

### Appendix A.2. Model Candidate Construction

A set of model candidates $C=\{c_{1},c_{2},\ldots ,c_{m}\}$ is constructed, where each candidate $c_{k}$ is defined by a quadruple:

$$c_{k}=\left({\mathrm{name}}_{k},E_{k},\Theta_{k},\sigma_{k}\right)$$

where ${\mathrm{name}}_{k}$ is the model identifier, $E_{k}$ is an unfitted scikit-learn-compatible estimator, $\Theta_{k}$ is a dictionary mapping pipeline parameter names to lists of candidate values, and $\sigma_{k}\in \left\{\mathrm{True},\mathrm{False}\right\}$ indicates whether numeric feature standardization is required.

The default candidate set includes LR, RF, MLP, XGBoost, LightGBM, and CatBoost.

**Algorithm A1** C-AutoML program.
**Require:** Data file path $p_{\mathrm{data}}$, target column *t*, iterations *n*, random seed $s_{\mathrm{base}}$, test proportion τ
**Ensure:** Optimal pipeline $P^{*}$ and associated output artifacts
1: $D\leftarrow \mathrm{ReadCSV}(p_{\mathrm{data}})$ 2: $t_{\mathrm{exact}}\leftarrow \mathrm{ResolveTargetColumn}\left(D,t\right)$ 3: $y,M\leftarrow \mathrm{EncodeBinaryTarget}(D\left[t_{\mathrm{exact}}\right])$ 4: $X\leftarrow D\setminus \{t_{\mathrm{exact}}\}$ 5: $C,S_{\mathrm{skipped}}\leftarrow \mathrm{BuildCandidates}\left(s_{\mathrm{base}}\right)$ 6: R←∅ ▹ Results container 7: **for** i←1 **to** *n* **do** 8: $s_{\mathrm{split}}\leftarrow s_{\mathrm{base}}+i$ 9: $X_{\mathrm{train}},X_{\mathrm{val}},y_{\mathrm{train}},y_{\mathrm{val}}\leftarrow \mathrm{TrainTestSplit}\left(X,y,\tau ,s_{\mathrm{split}}\right)$ 10: **for** $c_{k}\in C$ **do** 11: $s_{\theta }\leftarrow s_{\mathrm{base}}+i\;\cdot \;100+k$ 12: $\theta \leftarrow \mathrm{SampleParameters}(\Theta_{k},s_{\theta })$ 13: $\Pi \leftarrow \mathrm{BuildPreprocessor}(X_{\mathrm{train}},\sigma_{k})$ 14: $P\leftarrow \mathrm{Pipeline}(\Pi ,E_{k},\theta )$ 15: $P.\mathrm{fit}(X_{\mathrm{train}},y_{\mathrm{train}})$ 16: $\hat{y}\leftarrow P.\mathrm{predict}\left(X_{\mathrm{val}}\right)$ 17: $\tilde{y}\leftarrow \mathrm{GetScoresForAUC}\left(P,X_{\mathrm{val}}\right)$ 18: $m\leftarrow \mathrm{ComputeMetrics}(y_{\mathrm{val}},\hat{y},\tilde{y})$ 19: $R\leftarrow R\cup \{m,\theta ,c_{k}.\mathrm{name},i\}$ 20: **if** $m>m_{\mathrm{best}}$ ▹ Hierarchical comparison: $F_{1}$, ROC-AUC, accuracy **then** 21: $P^{*}\leftarrow P$ 22: $m_{\mathrm{best}}\leftarrow m$ 23: $X_{\mathrm{val}}^{*},y_{\mathrm{val}}^{*}\leftarrow X_{\mathrm{val}},y_{\mathrm{val}}$ 24: **end if** 25: **end for** 26: **end for** 27: $\mathrm{SaveArtifacts}(R,P^{*},X_{\mathrm{val}}^{*},y_{\mathrm{val}}^{*},S_{\mathrm{skipped}})$

### Appendix A.3. Preprocessing Strategy

A column-aware preprocessing pipeline is constructed for each trial:

- Numeric columns $X_{\mathrm{num}}$: Median imputation followed by optional StandardScaler when $\sigma_{k}=\mathrm{True}$.
- Categorical columns $X_{\mathrm{cat}}$: Mode imputation followed by one-hot encoding with handle_unknown = ‘ignore’.

The ColumnTransformer (scikit-learn’s column-routing preprocessor) is fitted exclusively on the training partition $X_{\mathrm{train}}$ to prevent data leakage into the validation set.

### Appendix A.4. Search Procedure

The algorithm executes *n* outer iterations, each using a unique stratified split with seed $s_{\mathrm{base}}+i$. For each iteration *i* and each model candidate $c_{k}$, a hyperparameter configuration θ is sampled from $\Theta_{k}$ using ParameterSampler with seed $s_{\mathrm{base}}+i\;\cdot \;100+k$. A full Pipeline is constructed and fitted:

$$\mathrm{pipeline}=\mathrm{Preprocessor}\circ E_{k}\left(\theta \right)$$

### Appendix A.5. Evaluation and Selection

For each trial, the validation set metrics $M_{\mathrm{val}}$ are computed:

(A1) $$\begin{array}{cc} \mathrm{Accuracy} & =\frac{TP+TN}{TP+TN+FP+FN} \end{array}$$

(A2) $$\begin{array}{cc} \mathrm{Precision} & =\frac{TP}{TP+FP} \end{array}$$

(A3) $$\begin{array}{cc} \mathrm{Recall} & =\frac{TP}{TP+FN} \end{array}$$

(A4) $$\begin{array}{cc} F_{1} & =2\;\cdot \;\frac{\mathrm{Precision}\;\cdot \;\mathrm{Recall}}{\mathrm{Precision}+\mathrm{Recall}} \end{array}$$

(A5) $$\begin{array}{cc} \mathrm{ROC-AUC} & =\int_{0}^{1}\mathrm{TPR}\left({\mathrm{FPR}}^{-1}\left(x\right)\right)\;dx \end{array}$$

where TPR and FPR denote true positive rate and false positive rate, respectively. The primary selection criterion is the $F_{1}$ score, with ROC-AUC and accuracy serving as tie-breakers in hierarchical order.

### Appendix A.6. Feature Importance

After the optimal pipeline $P^{*}$ is identified, permutation-based feature importance [15] is computed on the corresponding validation partition. For each feature $x_{j}$, the decrease in $F_{1}$ score is measured across r=10 independent shuffles:

$$I\left(x_{j}\right)=\frac{1}{r}\sum_{k=1}^{r}\left[F_{1}\left(P^{*},X_{\mathrm{val}}\right)-F_{1}\left(P^{*},X_{\mathrm{val}}^{\left(j,k\right)}\right)\right]$$

where $X_{\mathrm{val}}^{\left(j,k\right)}$ denotes the validation set with feature *j* permuted in shuffle *k*.

### Appendix A.7. Output Artifacts

The algorithm produces the following outputs in the specified directory:

- model_performance.csv: Complete trial results including all metrics and hyperparameters.
- best_model.joblib: Serialized optimal pipeline.
- model_characteristics.json: Comprehensive metadata including feature importance and per-model summaries.
- best_model_feature_effects.csv: Permutation importance per feature.
- best_model_confusion_matrix.csv and .png: Confusion matrix in tabular and visual formats.

### Appendix A.8. Required Python Packages

The implementation was developed in Python 3.9 and executed on Ubuntu 24.04.4 LTS. Dependencies were managed via Miniforge [36], a lightweight conda-forge-based distribution of Anaconda [37], with additional packages installed through pip [38] where conda-forge packages were unavailable. The following core packages are required:

- scikit-learn = 1.6.1—primary ML framework providing preprocessing transformers, supervised estimators, cross-validation utilities, and evaluation metrics [27].
- pandas = 2.3.1—tabular data ingestion, transformation, and feature engineering.
- numpy = 2.0.2—numerical array operations and statistical computations.
- matplotlib = 3.9.4—visualization of training curves, confusion matrices, and SHAP summary plots.
- joblib = 1.0.0—model serialization (.pkl artefacts) and parallelized cross-validation loops.

Optional packages enable extended gradient-boosting model support:

- xgboost = 2.1.4—scalable gradient-boosted trees [12].
- lightgbm = 4.6.0—histogram-based gradient boosting with reduced memory footprint [11].
- catboost = 1.2.8—gradient boosting with native handling of categorical features [13].
- shap = 0.49.0—model-agnostic explainability via SHapley Additive exPlanations [31].

## Appendix B. Preeclampsia Risk Prediction Web Interface and MLOPs Deployment

As a complementary contribution to the C-AutoML pipeline presented in this work, we developed a web-based inference interface and an MLOP deployment framework to operationalize the trained models within the computing infrastructure of the EPN. The system is currently accessible within the EPN campus network and exposes the best-performing models produced by the C-AutoML workflow and Amazon SageMaker Canvas procedure as a prediction service.

### Appendix B.1. MLOP Cycle and Experiment Tracking

Machine Learning Operations (MLOPs) refers to a set of practices that bring together machine learning development and software operations with the goal of deploying and maintaining models in production reliably and efficiently using MLflow [39] (=2.14.0). In this context, the MLOP cycle consists of four stages that repeat as new data becomes available: (i) training, where the C-AutoML workflow evaluates multiple model families and hyperparameter configurations; (ii) evaluation, where each trial is assessed using F1-score, accuracy, precision, recall, and AUC; (iii) registration, where MLflow stores the hyperparameter configuration, evaluation metrics, and serialized model artefact for every trial; and (iv) deployment, where the best-performing registered model is made available through the inference Application Programming Interface (API) consumed by the web interface.

MLflow acts as the tracking and model registry layer throughout this cycle. It records every C-AutoML trial as a named run under the experiment preeclampsia_severity, preserving a complete history of all model versions alongside their evaluation metrics. When a new training cycle produces a model that outperforms the currently deployed version, MLflow promotes that model to the serving stage. This promotion updates only the registry pointer that the inference API consults when loading the model at startup; the web interface and API code remain unchanged. The practical consequence is that the model serving the predictions can be improved or retrained as new data becomes available without any modification to the front-end application or the deployment infrastructure.

The model deployed in the current version of the system corresponds to the LightGBM model trained on the four demographic variables available from HGOIA, representing version 1 of the prediction service. This initial version is expected to evolve as the system collects or receives additional clinical data. When a richer dataset becomes available, the C-AutoML pipeline can be re-executed over the extended feature set, MLflow will register the new set of trials, and the best new model will automatically become the active version served to the interface. The current model therefore serves as the baseline deployment, and the architecture is designed to accommodate successive model generations without disruption to the clinical workflow.

### Appendix B.2. Data Capture Interface

The web interface, titled Verificador de Preeclampsia (Preeclampsia Risk Checker), allows a clinician to select a trained model and enter patient data through a structured form organized in clearly labeled sections. The input variables presented in the interface were designed to accommodate a broader set of clinical indicators than those available in the current study, in anticipation of richer data collection in future collaborations with health facilities. The active variable set used for inference is progressively adapted to the data effectively collected and provided by the participating institutions, so that the interface grows in clinical scope as the underlying dataset expands. Figure A1a–c illustrate the three scrollable sections of the interface.
